# Effect of Polymorphisms in *XPD* on Clinical Outcomes of Platinum-Based Chemotherapy for Chinese Non-Small Cell Lung Cancer Patients

**DOI:** 10.1371/journal.pone.0033200

**Published:** 2012-03-29

**Authors:** Wenting Wu, Huan Li, Huibo Wang, Xueying Zhao, Zhiqiang Gao, Rong Qiao, Wei Zhang, Ji Qian, Jiucun Wang, Hongyan Chen, Qingyi Wei, Baohui Han, Daru Lu

**Affiliations:** 1 State Key Laboratory of Genetic Engineering, Center for Fudan-VARI Genetic Epidemiology and MOE Key Laboratory of Contemporary Anthropology, Institute of Genetics, School of Life Sciences, Fudan University, Shanghai, China; 2 Beyster Center for Genomics of Psychiatric Diseases, Department of Psychiatry, University of California San Diego, La Jolla, California, United States of America; 3 Department of Respiratory Disease, Shanghai Chest Hospital, Shanghai Jiaotong University, Shanghai, China; 4 Division of Hematology and Oncology, Comprehensive Cancer Center, University of Alabama at Birmingham, Birmingham, Alabama, United States of America; 5 Department of Epidemiology, University of Texas M.D. Anderson Cancer Center, Houston, Texas, United States of America; University of Barcelona, Spain

## Abstract

**Purpose:**

Xeroderma pigmentosum group D (*XPD*) codes for a DNA helicase involved in nucleotide excision repair that removes platinum-induced DNA damage. Genetic polymorphisms of XPD may affect DNA repair capacity and lead to individual differences in the outcome of patients after chemotherapy. This study aims to identify whether *XPD* polymorphisms affect clinical efficacy among advanced non-small cell lung cancer (NSCLC) patients treated with platinum-based chemotherapy.

**Experimental Design:**

353 stage III-IV NSCLC patients receiving platinum-based chemotherapy as the first-line treatment were enrolled in this study. Four potentially functional *XPD* polymorphisms (*Arg^156^Arg*, *Asp^312^Asn*, *Asp^711^Asp* and *Lys^751^Gln*) were genotyped by matrix-assisted laser desorption/ionization time-of-flight mass spectrometry or PCR-based sequencing.

**Results:**

Variant genotypes of *XPD Asp^312^Asn, Asp^711^Asp* and *Lys^751^Gln* were significantly associated with poorer NSCLC survival (*P* = 0.006, 0.006, 0.014, respectively, by log-rank test). The most common haplotype GCA (in order of *Asp^312^Asn, Asp^711^Asp* and *Lys^751^Gln*) also exhibited significant risk effect on NSCLC survival (log-rank *P* = 0.001). This effect was more predominant for patients with stage IIIB disease (*P* = 2.21×10^−4^, log-rank test). Increased risks for variant haplotypes of *XPD* were also observed among patients with performance status of 0–1 and patients with adenocarcinoma. However, no significant associations were found between these polymorphisms, chemotherapy response and PFS.

**Conclusions:**

Our study provides evidence for the predictive role of *XPD Asp^312^Asn, Asp^711^Asp* and *Lys^751^Gln* polymorphisms/haplotype on NSCLC prognosis in inoperable advanced NSCLC patients treated with platinum-based chemotherapy.

## Introduction

Non-small cell lung cancer (NSCLC) accounts for approximately 80% of primary lung cancers, with most of the patients diagnosed at the advanced stage (stage III or IV) [1]. Although chemotherapy involving platinum agents represents one of the most common first-line treatments for these patients [2], the 5-year survival rate remains less than 15% [3]. Clinical factors, such as patient features (age and performance status) and disease extension, roughly characterized by the stage, remained the major determinations for prognosis of NSCLC [4]. However, variability in outcomes has still been observed in patients with similar clinical features. It seems that host inherited factors may have an important role in the determination of treatment outcome for NSCLC, which highlights the necessity of identifying genetic markers for optimally individualized therapy [5].

Considering that platinum agents used for chemotherapy cause DNA damage or cell death by activating the cell signaling pathways [6], the host cellular DNA repair capacity possibly influences the outcome of NSCLC patients after chemotherapy. Previous studies have shown that suboptimal DNA repair capacity within the tumor may lead to a decreased removal of DNA lesions, and therefore increase the sensitivity to platinum-based chemotherapy, resulting in better clinical outcome [7]–[9].

Nucleotide excision repair (NER) is the major pathway for repair of platinum-induced DNA cross-links in mammalian cells [10]. The *XPD* gene encodes for an ATP-dependent helicase, which mediates DNA unwinding for the initiation of NER [11]. Several studies have shown that *XPD* polymorphisms appear to have an effect on DNA repair capacity possibly by altering the function of protein product [12], [13]. Common variants in the *XPD* gene were found to be correlated with decreased cancer risk [14], [15]. And clinical and epidemiology evidences further indicate that the *XPD Lys^751^Gln* polymorphism was significantly associated with chemotherapy effect [16], [17]. However, for polymorphisms of *XPD Arg^156^Arg* and *XPD Asp^711^Asp*, epidemiologic data on clinical outcomes of NSCLC patients are still scarce. Therefore, the prognostic importance of *XPD* polymorphisms remains unclear.

The *XPD Asp^312^Asn* (rs1799793) and *Lys^751^Gln* (rs13181) polymorphisms are nonsynonymous single nucleotide polymorphisms (SNPs), resulting in a change to the amino acid sequence of protein. Whereas, the *Arg^156^Arg* (rs238406) and *Asp^711^Asp* (rs1052555) polymorphisms are both silent mutations. Our previous study has shown that variant genotypes of *XPD Arg^156^Arg* were associated with an increased risk of serious hematologic toxicity in Chinese Han population [18].

Based on these observations, we hypothesized that these four potentially functional SNPs of *XPD* (at codons 156, 312, 711 and 751) may influence NSCLC prognosis. In this study, using DNA samples obtained from a series of NSCLC patients, we assessed the association between *XPD* polymorphisms and survival in advanced NSCLC patients treated with platinum-based chemotherapy.

## Materials and Methods

### Patients recruitment and follow-up

The study design and subjects recruitment have been described previously [18]. Patients with inoperable and histologically confirmed stage III to IV NSCLC were consecutively recruited between March, 2005, and August, 2008, from Shanghai Chest Hospital in Shanghai, China. The eligible patients for this study were at least 18 years old and were required to fulfill the following criteria: the presence of a measurable lesion; Eastern Cooperative Oncology Group performance status (ECOG PS) 0∼2 (The Eastern Cooperative Oncology Group Performance Status are criteria used to assess how the disease affects daily life ability of patients by grading it into 5 grades.) [19]; an absolute neutrophil count (ANC) ≥1.5×10^9^ cells/L; platelets ≥100×10^9^ cells/L; serum creatinine ≤1.5×upper limit normal; Aspartate aminotransferase (AST) and alanine aminotransferase (ALT) ≤1.5×upper limit normal; estimated creatinine clearance ≥60 mL/min. Patients were excluded if they had a prior history of malignancy except for non-melanoma skin cancer, carcinoma *in situ* of the uterine cervix, or an already cured tumor (>5-y disease-free survival); previous chemotherapy, radiotherapy or surgery; active congestive heart failure, cardiac arrhythmia, or recent (<3 mo before the date of treatment) myocardial infarction; any severe mental disorder; infectious disease needing immunotherapy. A written informed consent was provided by each patient. The study was carried out with the approval of the Ethical Review Committee of the hospital.

Before treatment, all the patients received evaluation including complete medical history, health examination, scoring performance status, routine clinical biochemistry tests, chest radiographs and computed tomography of the chest and abdomen. Data on demographic and clinical characteristics (including age, sex, smoking status, and tumor histology) were obtained from clinical medical records with review by the oncologists. For smoking status, those who had smoked less than one cigarette per day and less than 1 year in their lifetime were defined as non-smokers, otherwise they were considered smokers. Information on survival statistics were collected from several sources, including follow-up calls, Social Security Death Index, and inpatient and outpatient clinical medical records. All the investigators were blind to the genetic polymorphism status of patients.

All the patients enrolled in this study were inoperable and received the first-line platinum-based chemotherapy. The chemotherapeutic regimens were as follows: navelbine (25 mg/m^2^) on day 1 and day 8 plus cisplatin (75 mg/m^2^) or carboplatin (AUC 5) on day 1, repeated every 3 week; gemicitabine (1,250 mg/m^2^) on day 1 and 8 plus cisplatin (75 mg/m^2^) or carboplatin (AUC 5) on day 1, repeated every 3 weeks; Taxol (175 mg/m^2^) plus cisplatin (75 mg/m^2^) or carboplatin (AUC 5) on day 1, repeated every 3 weeks; docetaxel (75 mg/m^2^) plus cisplatin (75 mg/m^2^) on day 1, repeated every 3 weeks. Few patients were given other platinum-based chemotherapy. All chemotherapy drugs were administered intravenously, and all treatments were for two to six cycles. Patient responses to the treatment were evaluated after first-line cycles of chemotherapy by the Response Evaluation Criteria in Solid Tumors (RECIST) guidelines version 1.0.

A total of 445 patients with advanced NSCLC participated in this study, of which 353 had been completely followed-up. No statistically significant difference was observed in the distribution of demographic and clinical features between the patients included in this study and those who did not (data not shown). As a result, our final analysis was limited to these 353 patients.

### SNP Genotyping

Blood samples were collected from each subject at the time of recruitment, and the genomic DNA was extracted using QIAamp DNA Maxi kit (Qiagen GmbH). The *XPD Arg^156^Arg*, *Asp^312^Asn*, and *Asp^711^Asp* polymorphisms were determined using the Sequenom MassARRAY iPLEX platform by the matrix-assisted laser desorption/ionization time-of-flight mass spectrometer as previously described [18], and the *Lys^751^Gln* was identified by PCR-based sequencing. Primer sequences are available on request. Overall, >98% of the genotypes were successfully determined for all the SNPs. For quality control, 5% samples of the subjects were randomly selected to re-genotype. All duplicate samples had a genotype concordance of 100%.

### Statistical analysis

Hardy-Weinberg equilibrium was tested for each SNP by goodness-of-fit X^2^ test. Pearson X^2^ test (for categorical variables) or student's t-test (for continuous variables) was used to compare the demographic and clinical characteristics. Patients achieving complete response (CR) or partial response (PR) were defined as “responders”, and patients with stable disease (SD) or progressive disease were defined as “non-responders”. Additional analyses were done by grouping patients with CR, PR, and SD (defined as “patients with clinical benefit”) versus those with PD (defined as “patients without clinical benefit”).

Overall survival (OS) was calculated as the time to death from the date of diagnosis. Progression-free survival (PFS) was calculated as the time to progression or death without progression from the date of diagnosis. Kaplan-Meier method was used to plot PFS or OS curve, and the log-rank test was applied to compare the distribution between groups. Multivariate Cox proportional hazards models were used to estimated hazard ratios (HR) with 95% confidence intervals (95% CI). Clinical variables with log-rank *P*<0.05 in univariate analysis were entered into multivariate analysis. Due to the limited number of homozygous variant genotypes, *Asp^312^Asn, Asp^711^Asp* and *Lys^751^Gln* polymorphisms were modeled in a dominant model. For linkage disequilibrium (LD) analysis, D′ and r^2^ values for the genotyped SNPs were calculated by Haploview software (http://www.broad.mit.edu/personal/jcbarret/haplo/). The haplotype assessment was performed using PHASE 2.0. Data were analyzed using SPSS, version 15.0 (SPSS, Inc.). All P values were two sides, and statistical significance was set at 0.05 levels.

To evaluate the chance of obtaining a false-positive association due to multiple hypothesis testing, we used the Bayesian false-discovery probability (BFDP) test [20] to calculate the probability of no association with a moderate range of prior probabilities 0.1 and 0.05 on the presence of an association. The BFDP threshold was set to 0.8, where a false non-discovery rate is four times as costly as a false discovery.

## Results

### Patient characteristics and clinical outcomes

Demographic and clinic features of patients are presented in Table 1. The median age was 57 (range, 32–80 years), and 246 (69.7%) patients were male. Among the subjects, 34 (9.6%) had stage IIIA disease, 107 (30.3%) had stage IIIB, and 212 (60.1%) had stage IV disease. Considering the histopathological type, 213 (60.3%) patients were diagnosed with adenocarcinoma, 75 (21.2%) with squamous cell carcinoma, 12 (3.4%) with adenosquamocarcinoma, and 53 (15.0%) with other carcinoma.

**Table 1:** Clinical characteristics of NSCLC patients.

Patients characteristics	n (%)
Total no. of patients	353
Median age (range)	57 (32–80)
Gender	
Male	246 (69.7)
Female	107 (30.3)
ECOG PS	
0–1	336 (95.2)
2	17 (4.8)
TMN stage	
IIIA	34 (9.6)
IIIB	107 (30.3)
IV	212 (60.1)
Histological type	
Adenocarcinoma	213 (60.3)
Squamous cell	75 (21.2)
Adenosquamocarcinoma	12 (3.4)
Others*	53 (15.0)
Smoking Status	
Never smokers	154 (43.6)
Ever smokers	199 (56.4)
Chemotherapy regimens	
Platinum- navelbine	187 (53.0)
Platinum-gemcitabine	54 (15.3)
Platinum- paclitaxel	73 (20.7)
Platinum- docetaxel	18 (5.1)
Other platinum combinations	20 (5.7)

NOTE: ECOG PS, Eastern Cooperative Oncology Group performance status; TNM, tumor-node-metastasis.

* Others include mixed cell, neuroendocrine carcinoma, or undifferentiated carcinoma.

### Association between polymorphisms and clinical benefit

Genotype information is presented in Table 2. All the genotype distributions were in accordance with Hardy-Weinberg equilibrium (*P*>0.05). Associations between polymorphisms and the efficacy outcome of response rate and clinical benefit were assessed using Pearson X^2^ test and univariate logistic regression. No SNPs were significant predictors of response rate or clinical benefit overall (Table 2).

**Table 2:** XPD Polymorphisms and Tumor Response by Univariate Analysis.

	Clinical Benefit (CR+PR+SD) (n = 286)				Response Rate (CR+PR) (n = 62)			
Polymorphisms	No. (%)	OR‡	95%CI‡	P†	No. (%)	OR‡	95%CI‡	P†
*Arg156Arg*								
C/C	73 (20.7)	1.00	Reference	0.775	18 (5.1)	1.00	Reference	0.682
C/A	154 (43.6)	1.12	0.52–2.40		31 (8.7)	1.31	0.68–2.51	
A/A	57 (16.1)	0.82	0.30–2.24		13 (3.6)	1.07	0.48–2.39	
*Asp312Asn*								
G/G	251 (71.1)	1.00	Reference	0.597	52 (14.7)	1.00	Reference	0.305
A/G+A/A	30 (8.5)	1.29	0.50–3.29		9 (2.5)	0.66	0.29–1.48	
*Asp711Asp*								
C/C	258 (73.1)	1.00	Reference	0.849	54 (15.3)	1.00	Reference	0.339
C/T+T/T	28 (7.9)	0.90	0.30–2.70		8 (2.3)	0.66	0.28–1.55	
*Lys751Gln*								
A/A	249 (70.5)	1.00	Reference	0.651	52 (14.7)	1.00	Reference	0.440
A/C+C/C	32 (9.1)	1.24	0.49–3.17		9 (2.5)	0.73	0.32–1.63	

NOTE: CR, complete response; PR, partial response; SD, stable disease; OR, odds ratio.

† Computed by Pearson Chi-Square test.

‡ estimated using univariate logistic regression.

### Association between *XPD* polymorphisms/haplotypes, PFS and OS

Table 3 shows PFS and OS analysis data according to examined polymorphisms. Overall median PFS was 7.3 months (95% confidence interval, 6.52–8.14 months). Although *Lys^751^Gln* polymorphism showed marginal correlation with PFS, there were no significant differences in PFS with respect to *XPD* genotypes.

**Table 3:** PFS and OS according to XPD polymorphisms.

	Patients				
Polymorphisms	No. (%)	Median PFS, mo (95% CI)	Log-rank *P*	Median OS, mo (95% CI)	Log-rank *P*
*Arg156Arg*					
C/C	85 (24.4)	7.5 (4.6–10.3)	0.691	18.0 (14.3–21.7)	0.759
C/A	194 (55.6)	7.7 (5.8–9.7)		19.0 (16.3–21.7)	
A/A	70 (20.1)	7.0 (5.9–8.0)		17.0 (14.4–19.6)	
*Asp312Asn*					
G/G	308 (89.0)	7.4 (6.3–8.5)	0.790	19.0 (16.7–21.3)	0.006
A/G+A/A	38 (11.0)	7.3 (4.9–9.8)		13.0 (10.2–15.7)	
*Asp711Asp*					
C/C	319 (90.4)	7.5 (6.4–8.6)	0.245	19.0 (16.6–21.4)	0.006
C/T+T/T	34 (9.6)	6.2 (2.0–10.4)		14.0 (11.1–16.9)	
*Lys751Gln*					
A/A	304 (86.1)	7.5 (6.0–8.9)	0.062	19.0 (16.3–21.7)	0.014
A/C+C/C	43 (12.2)	5.3 (3.5–7.0)		15.0 (13.0–17.0)	

NOTE: PFS: progression-free survival; OS: overall survival; CI: confidence interval.

**Figure 1:**
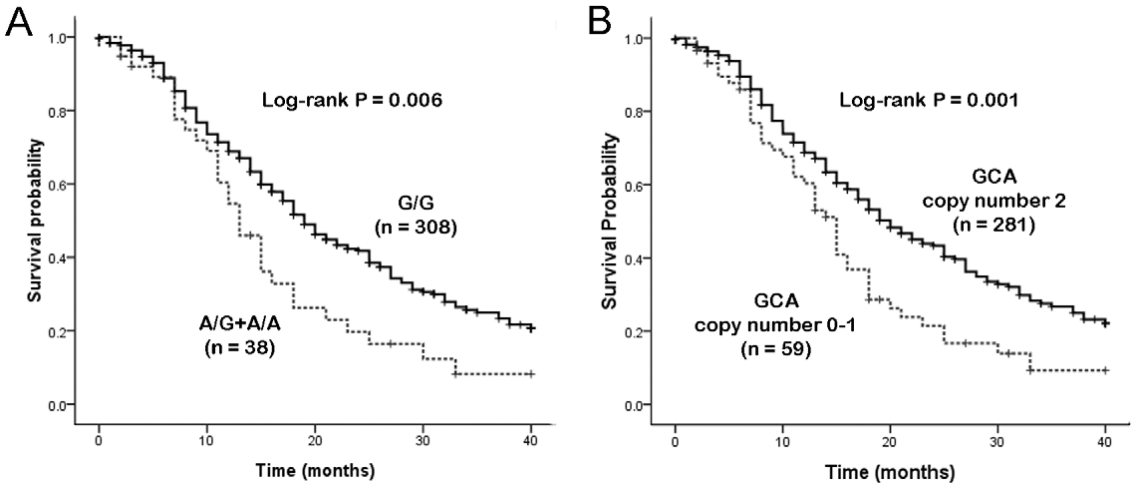
Kaplan–Meier curve of overall survival according to (A) *XPD Asp^312^Asn* polymorphism and (B) *XPD* haplotype.

*Asp^312^Asn*, *Asp^711^Asp* and *Lys^751^Gln* polymorphisms showed statistically significant associations with OS by log-rank test (Table 3, Table 4 and Fig. 1). For *XPD Asp^312^Asn* and *Lys^751^Gln* polymorphisms, the median survival time for patients with variant genotypes (13 months, 15 months, respectively) was shorter than that with wild-type homozygotes (MST, 19 months; log-rank *P* = 0.006, 0.014, respectively). These significances remained after adjustment for performance status, stage and treatment regimens (factors that were significantly associated with OS by log-rank test, Table S1) in the Cox regression model (adjusted *P* = 0.032, 0.034, respectively) (Table 4). For *Asp^711^Asp* polymorphism, the T-carrier genotypes were associated with reduced survival compared to the wild-type *C/C* genotype (14 months versus 19 months, log-rank *P* = 0.006). A marginally significant *P* value (0.053) was observed after adjustment for clinical features (adjusted HR, 1.54; 95% CI, 1.00–2.38). No significant difference between *Arg^156^Arg* and OS was observed.

**Table 4:** Polymorphisms associated with OS.

	Median OS,		Multivariate analysis		BFDP prior	
Polymorphisms	mo (95% CI)	Log-rank *P*	Adjusted Hazard Ratio (95% CI)†	*P* †	0.1	0.05
*Asp312Asn*						
G/G	19.0 (16.7–21.3)	0.006	1.00 (reference)	0.032	**0.77**	0.88
A/G+A/A	13.0 (10.2–15.7)		1.55 (1.04–2.32)			
*Asp711Asp*						
C/C	19.0 (16.6–21.4)	0.006	1.00 (reference)	0.053	0.82	0.90
C/T+T/T	14.0 (11.1–16.9)		1.54 (1.00–2.38)			
*Lys751Gln*						
A/A	19.0 (16.3–21.7)	0.014	1.00 (reference)	0.034	**0.77**	0.88
A/C+C/C	15.0 (13.0–17.0)		1.54 (1.03–2.29)			
*XPD* Haplotype						
Haplotype GCA‡						
0 (Copy number, 2)	20.0 (16.8–23.2)	0.001	1.00 (reference)	0.008	**0.50**	**0.68**
1 (Copy number, 0–1)	15.0 (13.0–17.0)		1.59 (1.13–2.22)			

NOTE: OS: overall survival; HR, hazard ratio; CI: confidence interval. BFDP: Bayesian false-discovery probability. Bold figures are those that remained noteworthy at the 0.8 BFDP level, suggesting true associations.

† Data were estimated from Multivariate Cox proportional hazards models, with adjustment of patient characteristics with P<0.05 in univariate analysis (performance status, clinical stage, and type of treatment regimens).

‡ Haplotype was composed in order of *Asp^312^Asn*, *Asp^711^Asp* and *Lys^751^Gln*; GCA was the most common haplotype.

Pairwise linkage disequilibrium (LD) for the four SNPs was presented in Table S2. A moderate LD was observed among *Asp^312^Asn, Asp^711^Asp* and *Lys^751^Gln* variants (0.42<*D′*<0.86). The most common haplotype GCA (in order of *Asp^312^Asn, Asp^711^Asp* and *Lys^751^Gln*) was found to account for 90.5% of the studied populations. The survival of patients with two copies of GCA haplotype (wild type) was significantly longer than that of the patients with zero to one copy (Table 4).

The BFDP values for the observed associations were calculated at two levels of prior probabilities (0.1 and 0.05) (Table 4). At the prior probability of 0.1, two SNPs (*Asp^312^Asn* and *Lys^751^Gln*) and the haplotype remained noteworthy (BFDP≤0.8).

### Subgroup analysis of association between *XPD* haplotypes and overall survival

The effects of *Asp^312^Asn* haplotypes on NSCLC survival were further evaluated by subgroups analysis of stage, histological type, chemotherapy regimen and performance status (Table 5). A more obvious evidence of association between the haplotypes and OS was observed in stage IIIB patients (log-rank *P* = 2.21×10^−4^). The variant haplotypes were associated with significantly decreased survival (MST, 15 months; adjusted HR, 3.11; 95% CI, 1.66–5.81), compared to the wild-type GCA haplotype (MST, 20 months). Statistically significant differences were also observed in other subgroups (patients with ECOG performance status of 0–1 and patients with adenocarcinoma) (Table 5). Moreover, haplotype distribution did not differ significantly among NSCLC patients, considering clinical factors as histology type, clinical stage and PS (Table S3).

**Table 5:** Subgroup analysis of association between *XPD* haplotype and overall survival.

	Haplotype GCA†								
	0 (Copy number, 2)		1 (Copy number, 0–1)					BFDP prior	
Variables	Death/Patients	MST (months)	Death/Patients	MST (months)	Log-rank *P*	Adjusted HR (95% CI)‡	Adjusted *P* ‡	0.1	0.05
Total	340								
TNM stage									
III B	45/83	20.0	16/19	15.0	2.21×10^−4^	3.11 (1.66–5.81)	3.8×10^−4^	**0.18**	**0.31**
IV	104/166	17.0	29/38	13.0	0.085	1.39 (0.91–2.11)	0.131	/	/
ECOG PS									
0–1	153/265	20.0	45/58	15.0	2.35×10^−4^	1.71 (1.22–2.40)	0.002	**0.27**	**0.44**
Chemotherapy regimens									
Platinum–navelbine	91/154	21.0	18/25	15.0	0.216	1.41 (0.84–2.35)	0.194	/	/
Platinum–gemcitabine	25/41	19.0	9/13	13.0	0.276	1.41 (0.63–3.16)	0.405	/	/
Platinum–paclitaxel	31/55	18.0	11/14	18.0	0.176	1.60 (0.78–3.28)	0.198	/	/
Histologic type									
Adenocarcinoma	93/168	21.0	27/35	13.0	0.004	1.82 (1.17–2.85)	0.009	**0.56**	**0.73**
Squamous cell	37/61	19.0	10/12	18.0	0.283	1.24 (0.61–2.56)	0.553	/	/

NOTE: ECOG PS, Eastern Cooperative Oncology Group performance status; TNM, tumor-node-metastasis; MST, median survival time; HR, hazards ratio; BFDP, Bayesian false-discovery probability. Bold figures are those that remained noteworthy at the 0.8 BFDP level, suggesting true associations.

† Haplotype was composed in order of *Asp^312^Asn*, *Asp^711^Asp* and *Lys^751^Gln*; GCA was the most common haplotype.

‡ Stratified tests was estimated by Multivariate Cox proportional hazards models, with adjustment of patient characteristics with P<0.05 in univariate analysis (performance status, clinical stage, and type of treatment regimens).

BFDP was conducted for the stratified analysis using the prior probability of 0.1 and 0.05 (Table 5). The observed associations remained noteworthy even at the prior probability of 0.05.

## Discussion

This study evaluated whether common XPD polymorphisms would influence clinical outcomes of Chinese NSCLC patients treated with platinum-based chemotherapy. We found that the variant genotypes and haplotypes of *Asp^312^Asn*, *Asp^711^Asp* and *Lys^751^Gln* polymorphisms significant contribute to shorter survival time compared to corresponding wild-type homozygous genotypes. After adjusting for clinical factors, the *XPD* haplotypes were still significantly associated with OS, and this effect was more predominant for patients with stage IIIB disease. Additionally, our BFDP correction suggested that observed associations of those SNPs in the main effect and subgroup analysis were probably not due to false discoveries.

XPD is a member of a nine-subunit complex TFIIH that acts in NER, and its helicase activity is required for the initiation of NER process. Both *in vivo* and *in vitro* studies have indicated that variations in the *XPD* gene may influence clinical benefits of the platinum-based therapy. Polymorphisms of *XPD* codon 312 and 751 have been investigated in several studies [12]–[17], [21]–[27], but no consensus has been reached. The genetic variations at codon 312 and 751 are nonsynonymous polymorphisms, resulting in a change to the amino acid sequence of protein. In a previous study, Seker et al. [28] reported that lymphoblastoid cells carrying the variant A/A genotype at codon 312 displayed a higher apoptotic response to UV compared with those carrying the wild-type genotype. Besides, several studies suggested that *XPD* 751-Gln substitutions might produce significant conformational change to the protein [25], leading to a low number of chromatid aberration and decreased risk of suboptimal DNA repair [12]. Our results were consistent with previous experimental and epidemiologic findings that the variant genotypes were associated with more efficient DNA repair capacity in human tissues, resulting in decreased effect of cytotoxic chemotherapy. The Asp711Asp polymorphism is a common silent substitution. The Asp711Asp and Lys751Gln polymorphisms were in strong LD (D′ = 0.86), and the exact function of Asp711Asp has not been elucidated yet. It could possibly affect the stability of mRNA or influence the rate of translation by converting a high usage codon to a low usage one. Alternatively, it was biologically plausible that this correlation was mediated by linkage disequilibrium with Lys751Gln or some other potentially functional SNPs.

In our analysis, a more significant association was found with the reduced survival in the haplotype analysis. This effect was still significant after the adjustment for clinical factors (adjusted P = 0.008). The cumulative effect of XPD haplotype might be the result of the synergistic effect of each SNP [29]. Further in vitro/vivo studies based on the combination of these three loci would help investigate the true biological mechanisms. Survival difference of haplotype was more apparent in patients with stage IIIB disease, which was highly in accordance with the study of Gurubhagavatula et al [8]. One of the possible explanations is that individuals with stage IV disease may have accumulated too many mutations during the course of tumor growth, which may drive treatment resistance and overwhelm any genetic change that may have altered DNA repair capacity [8].

A recent meta-analysis pointed out that none of *Asp^312^Asn* and *Lys^751^Gln* alone was significantly associated with OS in NSCLC patients receiving platinum-based chemotherapy [30]. However, when only Asians population were included, for *Lys^751^Gln* polymorphism, only one study which included 108 patients was eligible for final OS analysis; and for *Asp^312^Asn*, no study was eligible for OS analysis. As minor allele frequencies for those three polymorphisms are considerably lower in Asian population than those in European populations [31]–[33], adequate sample size was needed to boost the power of this study. Therefore, examination of these SNPs in our Chinese population will also help elucidate the mechanism of interethnic differences in the outcome of patients after chemotherapy.

The statistically significant differences in OS for these SNPs were also observed in patients with adenocarcinoma. Since adenocarcinoma was the most common tumor type in Chinese population and tended to show worse prognosis [34], this finding was meaningful for effective prediction in a particular subgroup of lung cancer patients. However, the XPD status in tumor cells needed to be determined in tumor specimens. Although survival outcome differed across *XPD* genotypes, response to therapy was not different among those subgroups. It suggested that genetic factors responsible for short-term efficacy were likely to be different from those for OS [35].

Our study has several strengths. Compared to published studies, a relatively large number of advanced NSCLC patients treated with platinum-based chemotherapy as the first-line treatment were enrolled in this study. The relatively homogeneous treatment standard limited the potential confounding effect. Both patient recruitment and data collection were carried out without the knowledge of genetic status. In addition, the consistency of subgroup analysis indicated that our findings were likely to be biologically plausible.

Limitations of this work included its retrospective single-center design and lack of other DNA repair genes. Until being confirmed by multi-center prospective studies, results from this study should not be over-interpreted. Since the incidence of EGFR mutation was expected to be particularly high in East Asian population, clinical trials examining *XPD* SNPs in NSCLC patients with regard to EGFR mutation may help to clarify whether *XPD* polymorphisms or haplotype could act as a predictive marker of chemotherapy.

In conclusion, our results provided evidences for the predictive role of *XPD Asp^312^Asn, Asp^711^Asp* and *Lys^751^Gln* polymorphisms/haplotype on NSCLC prognosis among NSCLC patients treated with platinum-based chemotherapy, which suggested that these *XPD* polymorphisms might be used in risk assessment for chemotherapy effect prediction and individualized treatment optimization.

## Supporting Information

Table S1 **Comparison of overall survival according to clinical characteristics of patients.**
(DOC)

Table S2 **Linkage disequilibrium (D′ and r^2^) between SNPs in** ***XPD*** **.**
(DOC)

Table S3 **Haplotype distribution according to clinical factors.**
(DOC)
