# Supplementary material for: Effect of Polymorphisms in XPD on Clinical Outcomes of Platinum-Based Chemotherapy for Chinese Non-Small Cell Lung Cancer Patients
Source: PLoS One. 2012 Mar 29;7(3):e33200. doi: 10.1371/journal.pone.0033200 (PMC3315552; doi:10.1371/journal.pone.0033200)
Supplement: Table S1 — Comparison of overall survival according to clinical characteristics of patients. (DOC) [file pone.0033200.s001.doc]

**Table S1. Comparison of overall survival according to clinical characteristics of patients**

|  | n | | MST  (Months) |  |  |
| --- | --- | --- | --- | --- | --- |
| Patient characteristics | Patients | Deaths |  | log-rank *P* | HR (95%CI) |
| Total no. of patients | 353 |  | 18.0 |  |  |
| Age |  |  |  |  |  |
| ≤ 57 years-old | 177 | 101 | 20.0 | 0.052 | 1.00 (reference) |
| > 57 years-old | 176 | 120 | 16.0 |  | 1.29 (0.99-1.69) |
| Gender |  |  |  |  |  |
| Male | 246 | 158 | 18.0 | 0.981 | 1.00 (reference) |
| Female | 107 | 63 | 18.0 |  | 1.00 (0.74-1.34) |
| ECOG PS |  |  |  |  |  |
| 0-1 | 336 | 209 | 18.0 | 0.002 | 1.00 (reference) |
| 2 | 17 | 12 | 10.0 |  | 2.45 (1.36-4.40) |
| TNM stage |  |  |  |  |  |
| III A | 34 | 16 | 27.0 | 0.010 | 1.00 (reference) |
| III B | 107 | 66 | 18.0 |  | 1.82 (1.05-3.14) |
| IV | 212 | 139 | 16.0 |  | 2.13 (1.27-3.58) |
| Smoking |  |  |  |  |  |
| Never | 154 | 88 | 19.0 | 0.190 | 1.00 (reference) |
| Ever | 199 | 133 | 18.0 |  | 1.19 (0.91-1.56) |
| Histologic type |  |  |  |  |  |
| Adenocarcinoma | 213 | 128 | 18.0 | 0.411 | 1.00 (reference) |
| Squamous cell | 75 | 49 | 19.0 |  | 1.07 (0.77-1.48) |
| Adenosquamocarcinoma | 12 | 9 | 13.0 |  | 1.59 (0.81-3.13) |
| Others^*^ | 53 | 35 | 14.0 |  | 1.24 (0.85-1.80) |
| Chemotherapy regimens |  |  |  |  |  |
| Platinum–navelbine | 187 | 115 | 19.0 | 0.040 | 1.00 (reference) |
| Platinum–gemcitabine | 54 | 34 | 18.0 |  | 1.03 (0.70-1.51) |
| Platinum–paclitaxel | 73 | 46 | 18.0 |  | 1.14 (0.81-1.60) |
| Platinum–docetaxel | 18 | 9 | 27.0 |  | 0.61 (0.31-1.21) |
| Other platinum  combinations | 20 | 16 | 10.0 |  | 1.96 (1.16-3.31) |

NOTE: ECOG PS, Eastern Cooperative Oncology Group performance status; TNM, tumor-node-metastasis; MST, median survival time; HR, hazard ratio.

* Others include mixed cell, neuroendocrine carcinoma, or undifferentiated carcinoma.
