# Supplementary material for: Effect of Polymorphisms in XPD on Clinical Outcomes of Platinum-Based Chemotherapy for Chinese Non-Small Cell Lung Cancer Patients
Source: PLoS One. 2012 Mar 29;7(3):e33200. doi: 10.1371/journal.pone.0033200 (PMC3315552; doi:10.1371/journal.pone.0033200)
Supplement: Table S2 — Linkage disequilibrium (D′ and r2) between SNPs in XPD . (DOC) [file pone.0033200.s002.doc]

**Table S2.** Linkage disequilibrium (D´ and *r^2^*) between SNPs in *XPD*.

| SNPs | *Arg^156^Arg* | *Asp^312^Asn* | *Asp^711^Asp* | *Lys^751^Gln* |
| --- | --- | --- | --- | --- |
| *Arg^156^Arg* | — | 0.907 | 0.372 | 0.308 |
| *Asp^312^Asn* | 0.049 | — | 0.427 | 0.517 |
| *Asp^711^Asp* | 0.006 | 0.142 | — | 0.867 |
| *Lys^751^Gln* | 0.006 | 0.254 | 0.555 | — |

NOTE. D´ values are given above the diagonal; r2 values are given below the diagonal.
