# Supplementary material for: Effect of Polymorphisms in XPD on Clinical Outcomes of Platinum-Based Chemotherapy for Chinese Non-Small Cell Lung Cancer Patients
Source: PLoS One. 2012 Mar 29;7(3):e33200. doi: 10.1371/journal.pone.0033200 (PMC3315552; doi:10.1371/journal.pone.0033200)
Supplement: Table S3 — Haplotype distribution according to clinical factors. (DOC) [file pone.0033200.s003.doc]

**Table S3.** Haplotype distribution according to clinical factors

|  | Haplotype GCA^†^ | | |
| --- | --- | --- | --- |
| Clinical factors | 0 (Copy number, 2) | 1 (Copy number, 0-1) | *P*‡ |
| Total no. (340) | n | n |  |
| Histological type |  |  | 0.403 |
| Adenocarcinoma | 168 | 35 |  |
| Squamous cell | 61 | 12 |  |
| Adenosquamocarcinoma | 7 | 4 |  |
| Others^*^ | 45 | 8 |  |
| TMN stage |  |  | 0.186 |
| IIIA | 32 | 2 |  |
| IIIB | 83 | 19 |  |
| IV | 166 | 38 |  |
| ECOG PS |  |  | 0.325 |
| 0-1 | 265 | 58 |  |
| 2 | 16 | 1 |  |

NOTE: PS, performance status; TNM, tumor-node-metastasis.

* Others include mixed cell, neuroendocrine carcinoma, or undifferentiated carcinoma.

† Haplotype was composed in order of *Asp^312^Asn*, *Asp^711^Asp* and *Lys^751^Gln*; GCA was the most common haplotype.

‡ P values were calculated by Fisher's Exact Test.
